# Bereaved relatives’ experiences during the incurable phase of cancer: a qualitative interview study

**DOI:** 10.1136/bmjopen-2015-009009

**Published:** 2015-11-25

**Authors:** Marleen N Wijnhoven, Wim E Terpstra, Ronald van Rossem, Carolien Haazer, Nicolette Gunnink-Boonstra, Gabe S Sonke, Hilde M Buiting

**Affiliations:** 1 Department of Registry & Research, Netherlands Comprehensive Cancer Organization, Utrecht, The Netherlands; 2 Department of Clinical Psychology, VU University, Amsterdam, The Netherlands; 3 Department of Internal Medicine, Onze Lieve Vrouwe Gasthuis, Amsterdam, The Netherlands; 4 Department of Pulmonology, Reinier de Graafgroep, Delft, The Netherlands; 5 Department of Internal Medicine, Reinier de Graafgroep, Delft, The Netherlands; 6 Division of Medical Oncology, Netherlands Cancer Institute, Amsterdam, The Netherlands

**Keywords:** CHEMOTHERAPY, ETHICS (see Medical Ethics), MEDICAL ETHICS, ONCOLOGY, PALLIATIVE CARE

## Abstract

**Objective:**

To examine bereaved relatives’ experiences from time of diagnosis of incurable cancer until death with specific emphasis on their role in the (end-of-life) decision-making concerning chemotherapy.

**Design:**

Qualitative interview study.

**Setting:**

Hospital-based.

**Participants and methods:**

In-depth interviews with 15 close relatives of patients who died from non-small cell lung cancer or pancreatic cancer, using a thematic content analysis.

**Results:**

All relatives reported that patients’ main reason to request chemotherapy was the possibility to prolong life. Relatives reported that patients receiving chemotherapy had more difficulty to accept the incurable nature of their disease than patients who did not. They mostly followed the patients’ treatment wish and only infrequently suggested ceasing chemotherapy (because of side effects) despite sometimes believing that this would be a better option. Relatives continuously tried to support the patient in either approaching the death or in attaining hope to continue life satisfactorily. Most relatives considered the chemotherapy period meaningful, since it sparked patients’ hope and was what patients wanted. Cessation of chemotherapy caused a relief but coincided with physical deterioration and an increased caregivers’ role; many relatives recalled this latter period as more burdensome.

**Conclusions:**

Relatives tend to follow patients’ wish to continue or cease chemotherapy, without expressing their own feelings, although they were more inclined to opt cessation. They experience a greater caregiver role after cessation and their feelings of responsibility associated with the disease can be exhausting. More attention is needed to reduce relatives’ distress at the end of life, also to fully profit from this crucial form of (informal) healthcare.

**Strengths and limitations of this study**

- This is the first study that examined close relatives’ impact on chemotherapy decisions.
- This is the first study that examined close relatives’ experiences and needs throughout different stages of incurable cancer.
- This study only included close relatives of patient from general/teaching hospitals. Possibly, results might be somewhat different in other clinical settings (university hospital/cancer centre, other tumour types). However, such differences probably mainly relate to patient characteristics, whereas this study focuses on the relatives’ perspective.

## Introduction

Advanced stage pancreatic cancer and non-small cell lung cancer (NSCLC) are devastating diseases with median life expectancies of approximately 6 and 10 months, respectively (see online supplementary appendix 1). With chemotherapy, the goal is to delay and relieve tumour-related symptoms. Response rates to first-line (pancreas) and second-line (NSCLC) chemotherapy are, however, usually small and only modest improvement of survival is observed.^1^ ^2^

Being aware of a poor prognosis and the non-existence of effective therapies may generate acceptance of the approaching death.^3^ Previous studies, however, have shown that patients often wish further anticancer treatment to postpone having to face the inevitable.^4^ ^5^ Doctors may follow patients’ wish for further treatment, because of anecdotal prior experiences of a notable treatment success and because they do not want to take away the patients’ hope for a longer life.^6^ Doctors’ inclination to follow the patient's wish may be enforced by clinical trials focusing on life prolongation of new, more effective treatment in specific patient groups. Trials comparing anticancer treatment with best supportive care and/or that include patient-reported outcomes as a primary outcome measure are rare.^7^

Close relatives of a patient may assist in achieving a balanced view of pros and cons in the decision-making process surrounding chemotherapy. The relatives’ perspective as a proxy of the patient's perspective or as a measure of the quality of death and dying is frequently investigated.^8^ It has not previously been investigated as a way to achieve additional insight about relatives’ impact on chemotherapy decisions, although prior studies report about the presence of close relatives when such decisions are being made.^9^ ^10^ Moreover, close relatives’ experiences and needs throughout different stages of incurable cancer have not previously been investigated, although prior studies show that relatives of patients with cancer often report physical, social and emotional problems.^11^

Such ‘pregrieving’,^12^ especially in cancers with a short time span to death, can affect relatives’ ability to ‘be there’ for the patient and possibly, their ability to participate in chemotherapy decisions. Surprisingly, the relatives’ role and their personal experiences have not previously been studied. Accordingly, the main aim of our study was to examine experiences of close relatives of patients who died from pancreatic cancer or NSCLC from the time of diagnosis of incurable cancer until death. Underlying research questions subsequently focused on relatives’ role in the (end-of-life) decision-making and on their (unmet) personal needs during the incurable phase of cancer.

## Methods

### Design and setting

This qualitative interview study is part of a larger project that examines chemotherapy use in the incurable phase of cancer. Apart from the present qualitative study, the project also comprises a study that explored in-depth what is documented about chemotherapy use/decisions in the patient's hospital medical files. Qualitative interviews are particularly useful to explore personal ideas, as they enable respondents to address themes that researchers may not have anticipated. We recruited relatives of patients from two Dutch hospitals and who died at least 6 months ago. We defined close relatives as persons who were closely involved and provided a majority of the patient's care. We recruited relatives in the following preference order: partner, children and other persons closely involved. Since the majority of relatives were partners of the patient, we often refer to ‘the partner’ (instead of the patient) while writing about relatives’ experiences.

### Recruitment and sampling

We used a purposive design, implying that the sample selected only included people of interest.^13^ We selected patients from our medical record study being diagnosed with pancreatic cancer or NSCLC (Buiting HM, Brink M, Wijnhoven M, Lokker ME, *et al.* Personal communication, 2015). The contact details in the patient's medical record enabled us to approach the participants. Next, the oncologist/pulmonologist invited participants with an information letter explaining the study goals. A nurse specialist subsequently contacted participants by phone and asked if they wanted to participate. Two independent female interviewers conducted the interviews (HMB and Astrid Onderwater, MSc (AO) both researchers in social sciences). Both interviewers had experience in performing interviews about sensitive topics. The interviews took place at the participant's home; one participant preferred to be interviewed in the hospital. Since the interviewers were not previously involved in the care for the patient, and because they were familiar in creating an informal atmosphere during the interviews, it can be assumed that relatives spoke freely about their experiences. Five interviews were attended by MNW. The participants were told that the interview was strictly anonymous and that they could stop at any time. The participants were informed about the study goals of the study and they knew about previous qualitative work that was performed by HMB. Since the interviewers were not involved in the care for the patient, it can be assumed that relatives spoke freely about their experiences. A lay version of the results will be sent to the participants; they can contact the researcher to comment on/ask for clarification.

### Interviews

The interview process started in June (2013) and lasted till December (2013). The interviews were semistructured and lasted on average 78 min (±27 min) and were tape-recorded. After every interview, some field notes were made. A topic list was developed in collaboration with healthcare professionals to conduct the interviews. This list was elaborately discussed with AO before starting the first interviews. The topic list included background information (age, (religious) belief of the relative), the relatives’ experiences regarding patients’ quality of life, the provision of chemotherapy and experiences in the very last stage of life. We started every interview with some general questions about the deceased patient such as the patient's character and the patient's age. We subsequently requested the relatives to describe what had happened and how they themselves were feeling throughout the disease course. Next, we asked them about their treatment experiences (including goals and expectations about treatment) and whether they felt that they could support their partner. In our topic list, example questions and bullets helped us to ask similar and open-ended questions. If relatives started to talk about something else, we let them continue, especially if the topic was mentioned in our topic list in a later stage. We did not conduct repeated interviews among the same participant. We addressed all topics in every interview and interviewed until data saturation was reached, that is, the point where no new or relevant information emerges with respect to the study question. The moment data saturation was reached was discussed with the whole project group (HMB, MNW, WET, NG-B and CH). After every interview, some initial field notes were made. The interviews were transcribed verbatim by a professional transcriber; these transcripts were not returned to the patient. We abstracted patient and decision-making characteristics using a coding instrument to ensure similar coding across interviews (see online supplementary appendix 2).

### Analysis

All interviews were read, coded and analysed with qualitative research software (Atlas-ti V.6.1.12) using a thematic content analysis by MNW and HMB. We (HMB and MNW) individually read eight interviews in different time intervals to identify general themes, and subsequently, specific categories within the themes to check for interpreter consensus concerning the assignment of text fragments to major themes: for example, for the theme ‘Slow acceptance of the incurable nature of the disease’, we provided categories such as receiving chemotherapy or not; relatives’ involvement in treatment decisions; and patients’ experiences in this.^13^ We discussed these themes, until we achieved consensus. After reaching agreement, MNW always checked whether the interpretations were in line with the existing data. Second, specific categories within the themes were identified to check for interpreter consensus concerning the assignment of text fragments to major themes. Third, interpretations were checked whether they were in line with the existing data and the preliminary results were discussed in different multidisciplinary project groups with expertise in health sciences, ethics, psychology, oncology and nursing (HMB, MNW, WET, NG-B, CH). The analysis was ongoing, implying that HMB and MNW repeatedly checked and rephrased specific items in the topic list based on previous interviews. The derivation of themes therefore was an ongoing process. This led us to add hypothetical case scenarios about ceasing chemotherapy, and to focus on relatives’ personal needs and wishes in the final phase of life. A professional translator translated the quotes that we eventually chose to illustrate our results. A lay version will be sent to the participants; they can contact the researcher to comment on/ask for clarification.

### Ethical considerations

The study was approved by the MEC-U (Medical research Ethical Committees United) of one hospital; on the basis of this METC approval, the other hospital had no objections for the study either. All relatives signed a consent form before the start of the interview.

## Results

### Relative and patient characteristics

In total, 33 bereaved relatives were approached; 15 of them participated in the study. In three interviews, more than one relative participated. Of the 33 bereaved relatives, 8 could not be reached and 10 did not want to participate. Two of them explicitly stated that such an interview was too burdensome. The sample consisted of relatives with an average age of 59 years (SD±11 years). Half of them were women and about a third was religious. Most relatives were the partner of the patient (73%).

Of the patients discussed, 7 (47%) were diagnosed with pancreatic cancer and 8 (53%) with NSCLC (see online supplementary appendix 2). Ten (67%) patients had received first-line chemotherapy and 5 (33%) had not; 8 (53%) died at home, 3 (20%) died in the hospital and 4 (27%) died in a hospice. In one patient, death was the result of euthanasia. Initiating first-line chemotherapy could be doctor-driven and/or patient-driven. Reasons to cease chemotherapy were mainly doctor-driven.

### Qualitative findings

We identified three domains that provided deepened insight into how close relatives experienced the incurable phase of cancer: the (slow) process of accepting the incurable nature of the disease, the moment when chemotherapy started to become useless and the changing care roles during different stages of the disease.

## (Slow) acceptance of the incurable nature of the disease

All bereaved relatives reported that patients’ main reason to opt for chemotherapy was the possibility that chemotherapy could extend their life.

That was her life saver, as long as the chemotherapy continues she said [patient] “if I have it, I know I will go on for a little bit longer”. Relative 7 (Patient with NSCLC—chemotherapy)

Relatives reported that patients who were not eligible for chemotherapy relatively soon accepted their fate saying that ‘it is what it is’. Knowing that chemotherapy was no option generated clarity about the course of the disease. The same applied to patients who had to stop further chemotherapy quickly. Such ‘quick’ acceptance was sometimes difficult to comprehend for the partners/close relatives themselves.

And to the doctor [patient] “I have had a good life and there's nothing that can be done about it”. […]The way I see it is if maybe he had had a different disease and had chemotherapy treatment that you would probably worry about these things even more. Relative 3 (Pancreas patient—no chemotherapy)

In patients who received one or more cycles of chemotherapy, acceptance of the disease more slowly developed than in patients who did not. These patients were clearly searching for new life goals.

R: I have to say that during the first year [being incurable] that it was quite difficult sometimes, because er…I think that it was because she was thinking about it an awful lot, thinking that the end was coming…but over the course of time it did improve […].

I: Why is that?

R: Er, maybe she found peace of mind—thought something like “OK, there's nothing that can be done about it, so let's try and enjoy things while it is still possible”. Relative 7 (Patient with NSCLC—chemotherapy)

Relatives of patients who had received chemotherapy were differently involved in the decision-making process. In patients for whom chemotherapy was considered a last straw to hang on to, relatives often preferred to watch silently: they did not want to bother the patient with difficult discussions.

That [chemotherapy] is not my decision when all is said and done. That is up to him [patient] and to the doctor. That was my view anyway. Relative 11 (Pancreas patient—chemotherapy)

In contrast, in patients for whom the decision to choose treatment with chemotherapy was more rationally driven, relatives more actively participated in the decision-making process.

Because he [patient] said “We need the time, the time to get everything sorted out”. […] So then we [patient/partner] discussed about slowing the process down a bit. Relative 10 (Patient with NSCLC—chemotherapy).

## When chemotherapy is starting to become useless

As was the case with the initial start of chemotherapy, relatives kept supporting their partner in subsequent chemotherapy decisions. Their role was ambivalent: to prepare for the approaching death, but also, to keep hope alive to continue life satisfactory.

Well, we talked about it together [approaching death]. And then fear welled up again; I kept having to say to her, to support her by saying “Now listen, you are still having your chemotherapy treatment and we are going to go for it”. Relative 7 (Patient with NSCLC—chemotherapy)

Doctors did the same by reassuring patients and relatives with a step-by-step and monthly approach. They preferred to monitor on a regular basis instead of giving prognoses of half a year or more (with or without chemotherapy). They further explicitly told patients/relatives that they only wanted to provide ‘useful’ care.

He always said “If I do give the chemotherapy, then it has be useful. And not if it isn't worthwhile, not just to please you because you say you really want it, and if I know that it will only make you more ill”. Relative 7 (Patient with NSCLC—chemotherapy)

Doctors seldom opted to cease chemotherapy, except in situations where scan results clearly showed that chemotherapy had had no tumor effect and chemotherapy was considered ‘useless’. Patients seldom spoke about the option to cease (further) chemotherapy either. In two interviews, patients explicitly chose to stop themselves.

That she said “wait a minute, I want to think about it”. Because he was like…he immediately wanted to make an appointment for her to come [for chemotherapy]. Relative 9 (Patient with NSCLC—chemotherapy)

Since relatives clearly noticed that their partners deteriorated after having received chemotherapy for a certain amount of time, and that they did not tolerate chemotherapy the way they did before, they sometimes started a discussion themselves about the possibility to cease chemotherapy.

I: And could you talk about it with him?

R: Yes…[…] but anyway, in January…it gradually became obvious that he was going to get another course of chemotherapy. And then I said to him, I said “XX are you sure you still want to do that”? And then he said “What do you mean, what should I do then”? I said “You could stop having treatment”. Relative 14 (Patient with NSCLC—chemotherapy)

Yet, patients often preferred and decided to continue chemotherapy and both patients and relatives generally followed the doctors’ advice. Although some of the relatives reported to have been disappointed about the way doctors (did not) discuss the severity of the prognosis and to be less specific about the treatment goals, they all trusted the doctor in choosing the right treatment regimen, partly because doctors stressed the importance to retain the patient's quality of life.

But the reason wasn't communicated clearly. He only said “I want your quality of life to be such that you are still able to enjoy everything.” But the exact reason why they stopped it [chemotherapy] was never made clear; it wasn't started again after that. Relative 8 (Patient with NSCLC—chemotherapy)

Most relatives often memorised the chemotherapy period as worthwhile despite side effects since this is what their partner had wanted. Still, the decision to discontinue chemotherapy nevertheless often appeared to be a relief.

So, yes, anyway it was so much better for her when in June or July we decided “That's it. We are going to stop now”. Relative 9 (Patient with NSCLC—chemotherapy).

## Changing care roles throughout different stages of the disease

When chemotherapy was stopped, physical deterioration necessitated that many patients still had to visit the hospital. In hindsight, these (frequent) hospital visits were often considered more burdensome compared with the chemotherapy period. Long waiting hours at the emergency department caused frustration and a feeling of powerlessness, partly because they realised that their partner/parent got worse every day.

Then there was a really hectic period when he always had something. […]. Every time they said “Well, you’ll have to go the Emergency Department” […] and there we were again waiting for four or five hours. It was really exhausting. Relative 14 (Patient with NSCLC—chemotherapy)

A large majority of relatives reported that being assertive was crucial in these situations, which was how they indeed behaved. Relatives reported that their role contrasted with the situation in which deciding about chemotherapy was still optional and in which the treating oncologist had final responsibility.

So I said to her “And you mean to tell me that […] is not possible?”. “Oh”, she said, “Oh, you're right, it is a lot of nonsense. I will get it organised.” Then I thought to myself “You really are left to their tender mercies if you can't stand up for yourself like I can”. Relative 10 (Patient with NSCLC—chemotherapy)

Although relatives seriously wanted to take care of their partner/parent to attain a high-quality meaningful life, at the same time they sometimes reported to regret that healthcare professionals paid little attention to their own necessities. Care could be extremely burdensome. Their personal wishes sometimes seemed to contrast with what the patient wanted. They, for instance, tried to prepare the most delicious meal, and make home as comfortable as possible, but they sometimes just could not satisfy the patient.

I bought all sorts of stuff, yoghurt—oh I don’t know—all sorts. And then he said to my son that he could just fancy a beer […] so there was a quick dash over the road to the supermarket. Relative 5 (Pancreas patient—no chemotherapy)

Care provided by volunteers or professional home care was not always sufficient or in accordance with their wishes. Psychosocial support, especially in later stages of the disease trajectory, was often given by general practitioners (GPs). GPs could be great coaches.

One Saturday someone came to wash him and I said “I'm just going out”. Really, I was so tired I could hardly put one foot in front of the other. I was totally exhausted. And then the GP contacted the hospice. Relative 5 (Pancreas patient—no chemotherapy)

Some of the relatives regretted that they had had no opportunities to openly speak about their partners’ approaching death, but most of the relatives did not. They accepted their partners’ wishes, which sometimes contrasted with what they themselves had wanted.

You know it's going to happen…in my head I was already working towards the end. He was maybe doing it unconsciously, but I certainly was. Relative 11 (Pancreas patient—chemotherapy)

When death approached, being together—either with (grand) children—significantly had increased in importance. Although relatives reported to experience this phase as rather burdensome, they at the same time reported that it had been an intense but precious time period.

…Sometimes it happens that someone dies suddenly, […] and then you haven't had that exhausting time, but at the same time you haven't had the time to say goodbye to each other. And the knowledge [that the disease is terminal], that is really tough and painful, it's really hard […] but…you are able to say goodbye to each other.

Relative 6 (Pancreas patient—chemotherapy).

## Discussion

Relatives purposefully tend to defer decisions about chemotherapy to the doctor. They describe their role as supportive, including support concerning patients’ struggles in accepting the incurable nature of the disease. They experience a more prominent role when chemotherapy is ceased: while contact/visits with the treating oncologist fall out and the condition of the patient deteriorates the greater responsibility concerning care provision can be exhausting.

Hobbs *et al* recently concluded that among approximately 5000 patients with lung and colorectal cancer, about half of them reported that their family members participated equally in their treatment decisions.^10^ They suggested that further studies were needed to determine the impact of family involvement. Our explorative study has direct relevance for ongoing discussions about the role of close relatives in patients with incurable cancer. Moreover, our findings provide new insight in how and when to assist the underserved group of close relatives from patients with incurable cancer to better recognise caregiver burden.^14^

### Strengths and weaknesses

Previous studies taking the bereaved relatives’ perspective as the starting point often focus on the last weeks/months of life. In our study, we focus on the disease trajectory from the moment patients/relatives had heard that the cancer could not be cured anymore. Our study also has limitations. First, the number of relatives is relatively small. However, since we achieved data saturation, no more in-depth interviews were needed. We interviewed relatives from patients who had been treated in general/teaching hospitals. Possibly, results might be somewhat different in other clinical settings (university hospital/cancer centre, other tumour types). However, such differences probably mainly relate to patient characteristics whereas this study focuses on the relatives’ perspective. Second, recall problems (eg, remember good memories and forget the worst ones since the latter persist longer) cannot be precluded since we present results of relatives, which were most often the partners. Third, although we did not collect detailed information, patients were usually over 60 years. It is plausible that relatives’ role is different and less passive in younger adults facing different challenges.^15^ Finally, doctors’ experiences are lacking in this study. Such an additional perspective could have been informative, for example, by comparing to what extent doctors experienced the same as relatives did.

### Close relatives: assisting in evaluating the patient's quality of life

Our study shows that chemotherapy is usually ceased when the doctor considers further treatment inappropriate (balancing the benefits and burdens). Few clinical studies using health-related quality of life (HRQoL) are available to doctors to guide such decisions. Moreover, HRQoL questionnaires may not even address the most important items since they mainly assess symptoms related to the physical consequences of the disease, whereas psychological and spiritual issues become far more important when being incurable.^16^

Our study shows that to achieve a meaningful high-quality life, relatives tried to support their partner to deal with the incurable nature of their disease. They seemed to struggle between either broaching discussions about the last stage of life and/or stimulating the patient to keep hope alive. In doing this, they usually did not speak about the approaching death, although they tried to enable thinking about the eventuality of life together. Our finding that patients’ main reason to opt for chemotherapy to possibly prolong their life is noteworthy since life prolongation by chemotherapy is often modest at most. Possibly, findings may be different in patients with incurable colorectal, prostate or breast cancer who usually have much longer disease trajectories.

In hindsight, it is difficult to determine whether burdensome treatment should have been omitted in these patients. Relatives, however, seldom experienced the chemotherapy period as too burdensome. They sometimes opted to cease chemotherapy to preserve the patient's quality of life. Our findings suggest that relatives do indeed have an important role in evaluating the patient's quality of life and so assist in evaluating the decision-making process about (further) chemotherapy.

### Close relatives: exhausted during the last stage of a patient's life

Our study further shows that relatives often experienced the period after chemotherapy was ceased to be more burdensome. At present, attention towards the caregiver burden that results from the provision of care for patients with chronic illness is increasing.^14^ In oncology, due to the availability of more treatment options, the time period in which relatives take care for patients with incurable cancer will increase in the near future. This time period may go hand in hand with an increase in hospital visits, including visits to emergency departments. It is frequently recommended that these hospitalisations should be avoided, especially in the very last stage of life.^17^ Our study showed that emergency visits were often requested by patients and followed by close relatives and doctors. Underlying reasons (including the absence of adequate professional home care) for hospital admittances therefore also need to be analysed in retrospective studies when drawing conclusions as to whether such hospital referrals should and can be avoided or not.

Yet, the accompanying distress of close relatives, especially in the last months of a patient's life, warrants further reflection. As has been shown in previous studies,^12^ ^18^ complicated grief (and subsequent health problems and distress) experienced by close relatives is common. Our study clearly showed that most of the relatives were distressed due to an increased caregiver burden as well as insufficient attention from healthcare providers towards their own necessities. It could be argued that if complicated grief prior to death is ‘tackled’ in advance, providing adequate end-of-life care may improve and at the same time be less burdensome for the relatives. Yet, our study showed that distress was especially raised in the final months (in the absence of chemotherapy), which suggests that preventing complicated grief prior to death has less impact on treatment decisions itself.

Apart from taking care for their personal needs, our study showed that professional home care could also be a relief. It however also limited the patient's and partner's autonomy while being together in this last period of their life. This is something which should be further explored. Dionne-Odom *et al*^19^ recently concluded that family caregivers of patients with advanced cancer experienced lower depression and stress burden with early (vs delayed) palliative care support that is also focusing on the family caregiver. Although this study focused on all patients with cancer with an estimated prognosis of 6–24 months and was conducted in a US setting, the results are novel and promising. On the basis of our qualitative findings, early integration of palliative care thus seems to combat with other dimensions of (patients’ and relatives’) distress than the previously mentioned grief prior to death and warrants further research.

## Conclusions

Our study shows that the perspective of close relatives has added value to optimise the (end-of-life) decision-making process, but that their personal needs remain somewhat invisible when compared with the patient. Offering appropriate (end-of-life) care, taking into account both the patient and relative perspective can be challenging.

- To explicate relatives’ role in the decision-making more study is warranted, also with respect to new forms of less toxic systemic treatments, which may influence the decision-making process.
- To provide appropriate end-of-life care more study is warranted, to relieve relatives’ distress, but also, to support relatives in their crucial role in the care for the patient.
